# Use of Tri-Ponderal Mass Index in Predicting Late Adolescent Overweight and Obesity in Children Aged 7–18

**DOI:** 10.3389/fnut.2022.785863

**Published:** 2022-03-21

**Authors:** Xijie Wang, Jun Ma, Sizhe Huang, Bin Dong, Yanhui Dong, Zhaogeng Yang, Jie Hu, Wannian Liang

**Affiliations:** ^1^Vanke School of Public Health, Tsinghua University, Beijing, China; ^2^Institute for Healthy China, Tsinghua University, Beijing, China; ^3^Institute of Child and Adolescent Health, School of Public Health, Peking University, Beijing, China; ^4^Zhongshan Health Care Center for Primary and Secondary Schools, Guangdong, China; ^5^Menzies Health Institute Queensland, Griffith University, Brisbane, QLD, Australia

**Keywords:** body mass index, tri-ponderal mass index, cohort study [or longitudinal study], obesity screening, child and adolescent

## Abstract

**Background:**

Current reference systems using body mass index (BMI) or BMI z-scores to estimate overweight and obesity risk in adolescents are complex to use. An easy and effective measure and cutoffs such as the tri-ponderal mass index (TMI) are in need for parents and grassroots health workers.

**Objective:**

The aim of this study was to test whether cohort-derived TMI could be efficient for obesity prediction and to find out whether simplified TMI cutoffs could be used in the prediction.

**Methods:**

Data were obtained from a 12-year retrospective growth cohort generated in Guangdong, China. A total of 17,815 children (53.9% were boys) with 151,879 follow-ups conducted annually between 2005 and 2016 were involved. Late adolescent overweight and obesity were defined based on the BMI z-score (WHO 2007 growth reference) of the last measurement, which happened at the mean age of 17.2 (SD: 0.7) for both sexes. Analysis of the area under the curve (AUC) of the receiver operating characteristic curves was used to find the most appropriate cutoff.

**Results:**

In total, 9,604 boys and 8,211 girls were included in the final analysis. TMI cutoffs performed better than WHO BMI cutoffs in the prediction of late adolescent overweight and obesity, with all corresponding AUCs <0.7. The simplified TMI cutoffs used to predict late adolescent overweight and obesity were 13.1 and 14.1 kg/m^3^ for children aged 7 to 15 years, respectively, with the corresponding AUCs ranging from 0.7315 (standard error, SE: 0.0132) to 0.9367 (SE: 0.0052). The cutoffs for predicting late adolescent overweight and obesity for children aged 16 to 18 years were 14.0 and 15.8 kg/m^3^, respectively, with the corresponding AUCs ranging from 0.9189 (SE: 0.0048) to 0.9841 (95% CI: 0.0027).

**Conclusion:**

Tri-ponderal mass index with the ease of administration in practice could be a promising alternative screening tool to BMI for the prediction of late adolescent overweight and obesity.

## Introduction

Obesity has been one of the major threats to public health worldwide (1, 2). According to the data of Global Burden of Diseases, Injuries, and Risk Factors Study (GBD) in 2015, China and India had the highest numbers of obese children (2). From the latest update of GBD 2019, high body mass index (BMI) has continued to be one of the most important risk exposures for human beings (3).

Apart from its confirmed relationship with various diseases, obesity has also been defined as a non-communicable disease by The Obesity Society in 2018 (4). It is widely accepted that children's overweight or obese status could track into their adulthood (5, 6) and could lead to a large burden to both individual health and the national medical system. A pooled meta-analysis found that children and adolescents with obesity were around five times more likely to be obese in adulthood than those who were not, and around 80% of adolescents with obesity would be obese in adulthood (7). Primary prevention that started in late childhood and continued during adolescence would be essential to avoid a wide range of adverse intergenerational effects caused by obesity (8).

For decades, BMI was a widely acceptable evaluation measure for obesity screening. However, the following limitations were noted persistently. First, the recommended BMI cutoffs for screening overweight and obesity in current guidelines were derived from cross-sectional data, including the WHO 2007 Growth Reference, the Chinese Screening for Overweight and Obesity among School-age Children and Adolescents (WS/T 586-2018, issued by the National Health Commission of P.R. China), and the Growth Chart developed by the Center of Disease Control for the United States (9–11). A recent study has found that BMI cutoffs generated from local cohorts could better predict overweight and obesity in early adulthood, despite the lack of the Asian population (12). Second, the stability of BMI in adolescents has been interrogated. It has been suggested that the appropriate scaling power of weight/height may fluctuate between 1.5 and 3.5 (13–16). The tri-ponderal mass index (TMI), which is calculated as weight/height^3^, is relatively stable during adolescence, and it has been suggested as an alternative measure to BMI for estimating body fat level and obesity-related metabolic risk (17–19). However, most of these relationships were identified in cross-sectional studies and were rarely confirmed in cohort settings.

In this study, with the school physical examination cohort in Zhongshan (Guangdong, China), we aim to (1) compare the capability of predicting late adolescent overweight and obesity using cohort-derived BMI cutoffs, cohort-derived TMI cutoffs, and WHO BMI reference and (2) examine whether simplified TMI cutoffs could be used for predicting late adolescent overweight and obesity.

## Methods

### Study Population

The data used for the present analysis were a 12-year retrospective cohort, which was built upon the Zhongshan school health examinations, which were conducted once a year from 2005 to 2016. All the children who entered primary school in 2005 were included in the dataset and have been described previously (20). In this analysis, 17,815 children (53.9% boys) with 151,879 follow-up measurements were included to generate sex- and age-specific cutoffs for predicting overweight and obesity. Participants were included if they (1) had at least 8 measurements with a full record of the date of examination, weight, and height and (2) had at least one measurement between the age of 16 and 18. Written authorizations by Zhongshan Health Care Center for Primary and Secondary Schools have been obtained for the use of the data. All personal information that could be related to identifying the specific child was removed. This analysis has been approved for exemption of ethical application by the Institutional Review Board of Peking University (IRB 00001052-20011-免).

### Study Measurements and Outcomes

All physical examinations were conducted by qualified medical physicians. Children's sex and date of birth were collected at their first physical examination in primary school and were recorded in the school registration system. The age (in year) at each follow-up was calculated as (date of examination—date of birth)/365.25.

Height was measured to the nearest 0.1 cm using the portable stadiometer (model TZG, Jiangyin No. 2 Medical Equipment Factory, Jiangsu Province, China), while students were standing straight with barefoot. Weight was measured to the nearest 0.1 kg using a lever-type weight scale (model RGT-140, Shanghai Dachuan Electronic Weighing Apparatus Co Ltd, Shanghai, China), while children were wearing undergarments.

Children's BMI at each follow-up was calculated as weight (kg)/height^2^ (m)^2^, whereas TMI was calculated as weight (kg)/height^3^ (m)^3^. Age- and sex-specific BMI z-scores were converted according to WHO 2007 growth reference (9), which were the most widely accepted indicators for overweight and obesity screening globally. Therefore, the BMI z-scores were set as the standard references in this study. Round-down integer age was used to generate age-specific BMI and TMI cutoffs. Overweight and obesity at late adolescence were defined based on the BMI z-score of the last measurement, which happened at the mean age of 17.2 (SD: 0.7) for both sexes.

### Statistical Analysis

A package named GAMLSS from R version 3.6.3 was used to generate smoothed age- and sex-specific TMI percentiles to explore the TMI trajectories between the ages of 7 and 18 years. First, ROC analysis and optimal cutoff point analysis were conducted for each sex and age group to derive the best fit age-specific BMI and TMI cutoffs, recorded as “BMI-cohort cutoffs” and “TMI-cohort cutoffs” (hereafter recorded as BMI-cohort and TMI-cohort), respectively, to discriminate overweight or obesity at late adolescence were determined by the maximum area under the curve (AUC) of the receiver operating characteristic (ROC) curves (21). Next, various regression models for age and TMI cutoffs were applied, including linear, quadratic, logarithmic, log-linear, and log-log models; the best fit models were selected based on adjusted R^2^ (alternative models were displayed in Supplementary eTable 1). To test the capability of calculated TMI cutoffs in predicting late adolescent overweight and obesity, the AUCs of age-specific TMI and BMI cutoffs were compared to BMI cutoffs recommended by WHO (9) (recorded as BMI-WHO cutoffs). Furthermore, we attempted to merge the TMI cutoffs across age and sex groups for easier memory and less calculation. The above procedure on how we get the final TMI cutoffs were displayed in Supplementary eFigure 2. TMI cutoff points for overweight and obesity risks were merged based on the distribution of age- and sex-specific TMI cutoffs, and the final TMI cutoffs were recorded as simplified TMI. TMIs were subsequently tested for sensitivity, specificity, and AUC with the test of equality of ROC curves and with TMI-cohort as reference. The best-fit cutoffs selected from each procedure were the ones with maximum AUC (≥0.7) and a lower false-negative rate for overweight and obesity risk.

Except for generating TMI centiles, all statistical analyses were performed with Stata version 14.0 (StataCorp LP, College Station, TX). Two-tailed *p-*value < 0.05 was considered statistically significant.

## Results

### BMI and TMI in Children From 7 to 18 Years

In total, 9,604 boys and 8,211 girls were involved in this analysis. The mean age at baseline was 9.0 (SD: 1.2) years, while the mean age at the last measurement was 17.2 (SD: 0.7) years. At the endpoint, the prevalence of overweight was 10.2% for boys and 5.3% for girls, while that of obesity was 3.3% for boys and 1.1% for girls (Table 1). Unlike BMI, TMI values remained relatively stable during adolescence, and the centile cutoffs fluctuated within a limited range. True values of BMI and TMI at each age are displayed in Supplementary eFigure 1 and Supplementary eTable 2.

**Table 1:** Participants' characteristics by sex.

**Variables**	**Boys**	**Girls**
Number of participants, *n* (%)	9,604 (53.9)	8,211 (46.1)
Baseline age, year, mean (SD)	9.0 (1.2)	9.0 (1.2)
Baseline BMI, kg/m^2^, mean (SD)	18.5 (3.5)	18.0 (3.2)
Baseline TMI, kg/m^3^, mean (SD)	12.4 (1.7)	11.9 (1.5)
Mean follow up time, year, mean (SD)	8.3 (1.2)	8.3 (1.2)
Age at endpoint*, year, mean (SD)	17.2 (0.7)	17.2 (0.7)
BMI category at endpoint*		
Normal weight, *n* (%)	8305 (86.5)	7686 (93.6)
Overweight, *n* (%)	982 (10.2)	432 (5.3)
Obesity, *n* (%)	317 (3.3)	93 (1.1)

SD, standard deviation; BMI, body mass index; TMI, tri-ponderal mass index. *Defined by the last measurement of each participant.

### Development of Age- and Sex-Specific TMI Cutoffs

**Figure 1:**
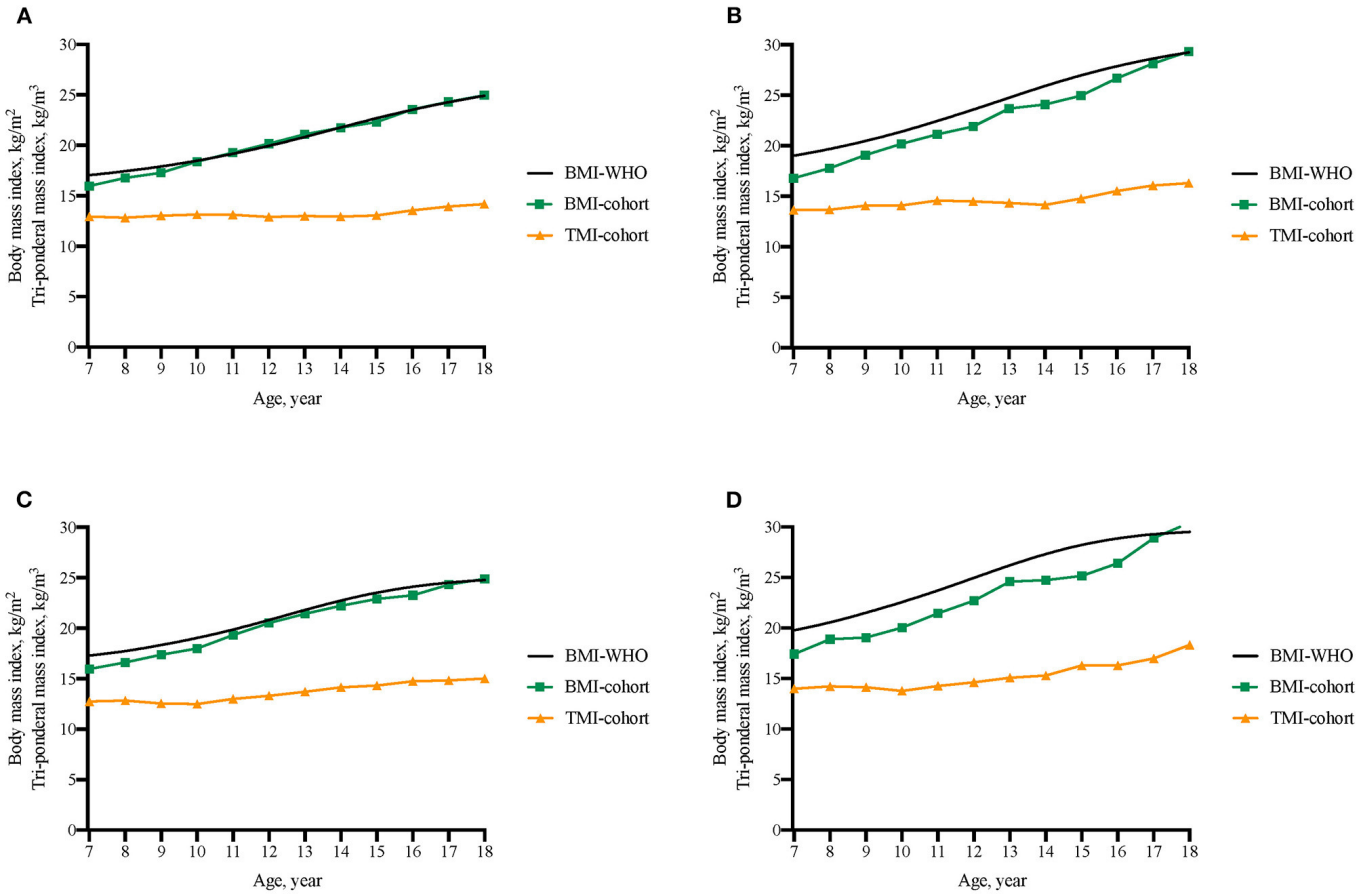
Comparison of BMI threshold from WHO 2007 reference (BMI-WHO), BMI cutoffs from the present cohort (BMI-cohort), and TMI cutoffs from the present cohort (TMI-cohort) to discriminate overweight and obesity in late adolescence. **(A)** Boys' cutoffs to predict late adolescent overweight; **(B)** Boys' cutoffs to predict late adolescent obesity; **(C)** Girls' cutoffs to predict late adolescent overweight; **(D)** Girls' cutoffs to predict late adolescent obesity.

However, as given in Supplementary eTables 3, 4, and Figure 1, the best-fit cutoffs of TMI (TMI-cohort) to discriminate late adolescent overweight and obesity were fluctuating with age. The age-specific BMI cutoffs to predict overweight at late adolescence derived from our cohort were close to the BMI value corresponded to BMI z-score of 1 from WHO growth reference and the BMI-cohort to predict obesity at late adolescence were slightly lower than the BMI cutoffs corresponded to BMI z-score of 2 from WHO reference. The sensitivity, specificity, and AUC of TMI-cohort were stable and relatively high across age and sex groups. The relationship between age and TMI cutoffs was also assessed using multiple regression models to explore if there was a fitting equation, and the results are shown in Supplementary eTable 5.

**Figure 2:**
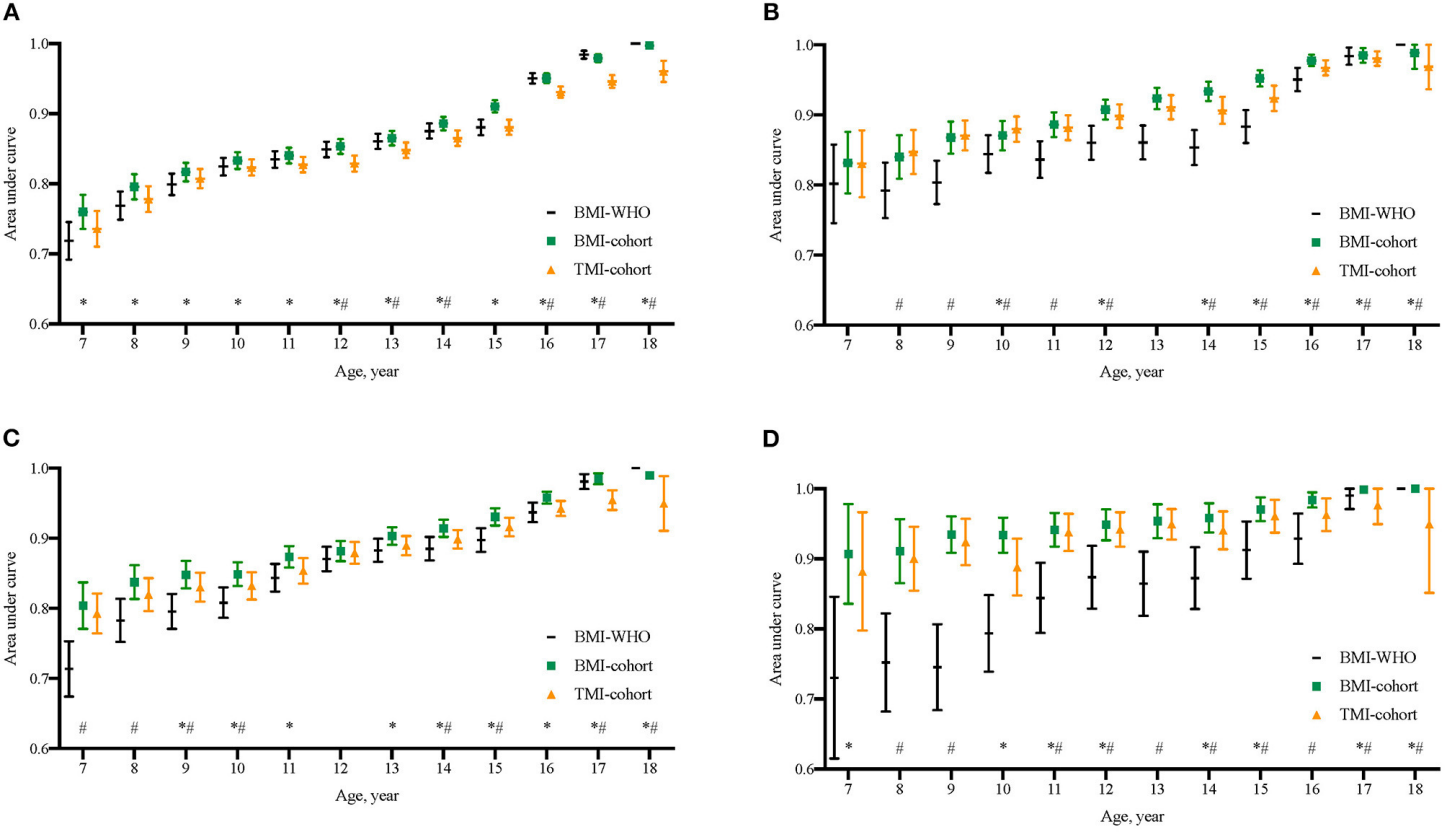
Comparison of area under curves (AUCs) of BMI cutoffs from WHO 2007 growth reference (BMI-WHO), BMI cutoffs from the present cohort (BMI-cohort), and the calculated TMI cutoffs from the present cohort (TMI-cohort) to discriminate overweight and obesity in late adolescence. **(A)** Boys' endpoint overweight; **(B)** Girls' endpoint overweight; **(C)** Boys' endpoint obesity; **(D)** Girls' endpoint obesity. *The AUC of BMI-cohort at that age was significantly different from that of TMI-cohort; ^#^The AUC of BMI-WHO at that age was significantly different from that of TMI-cohort.

In comparison with BMI-WHO cutoffs, both the calculated TMI cutoffs and BMI-cohort showed high AUC for predicting overweight and obesity at late adolescence, and the AUC increased significantly with age (Figure 2). In total, BMIs had the highest AUC in predicting late adolescent overweight and obesity and therefore provided a higher and more reliable reference standard for studying the performance of TMI cutoffs. To predict overweight in boys, the AUC of three cutoffs differed within a small range. For instance, the corresponding AUC for the three cutoffs at age 7 were 0.7187 (95% CI: 0.6919, 0.7454; BMI-WHO), 0.7599 (95% CI: 0.7356, 0.7842; BMI-cohort), and 0.7358 (95% CI: 0.7103, 0.7612; TMI-cohort), respectively. However, in terms of the ability in predicting overweight in girls and obesity in both sexes, both cohort BMI cutoffs and cohort TMI cutoffs performed better than BMI-WHO cutoffs, though BMI-WHO cutoffs caught up after the age of 15.

### The Development of Simplified TMI Cutoffs

**Figure 3:**
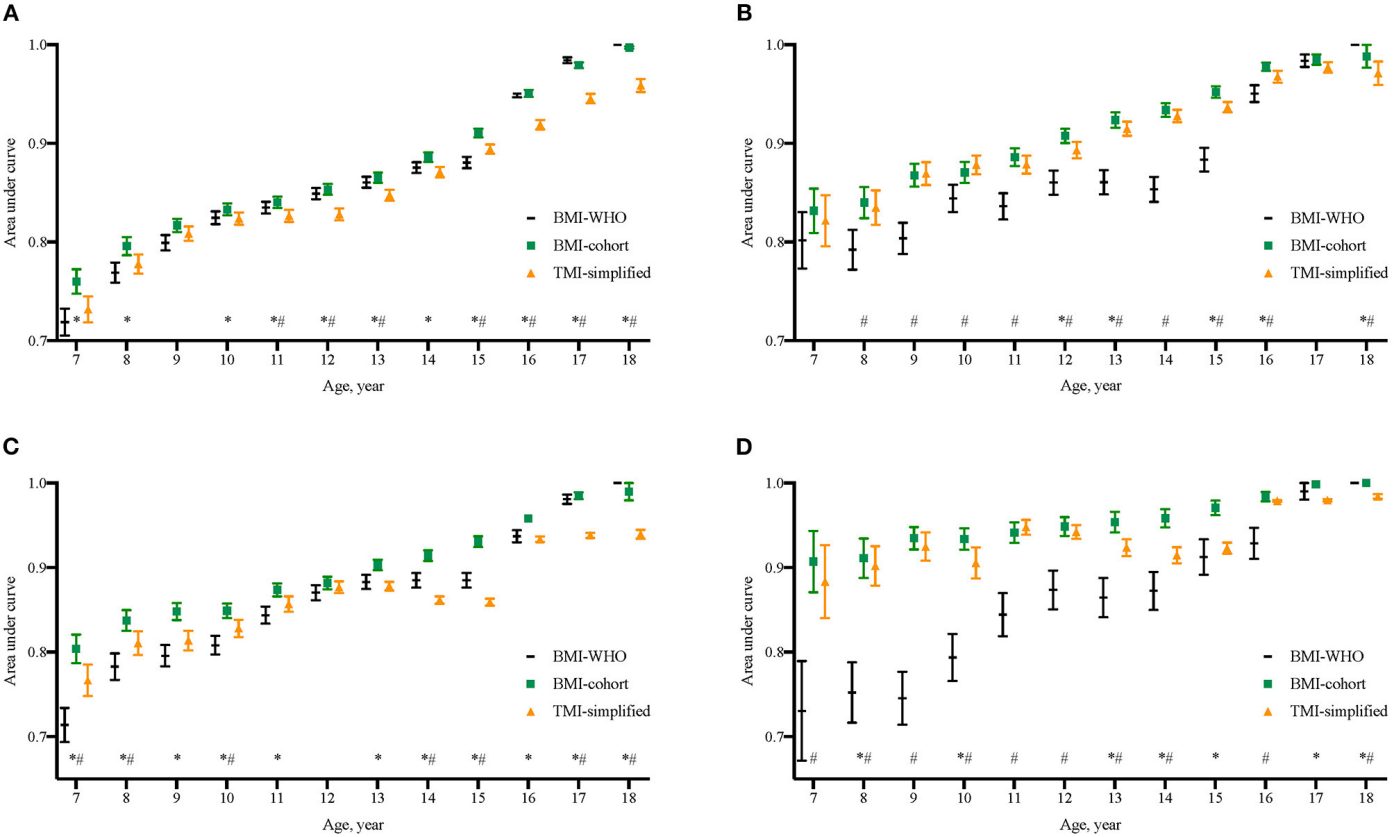
Comparison of area under curves (AUCs) of BMI cutoffs from WHO 2007 growth reference (BMI-WHO), BMI cutoffs from the present cohort (BMI-cohort), and the merged TMI cutoffs from the present cohort (TMI-simplified) to discriminate overweight and obesity in late adolescence. *The AUC of BMI-cohort at that age was significantly different from that of TMI-merged; ^#^The AUC of BMI-WHO at that age was significantly different Q20 from that of TMI-merged. **(A)** Boys' endpoint overweight, **(B)** Girls' endpoint overweight, **(C)** Boys' endpoint obesity, and **(D)** Girls' endpoint obesity.

**Figure 4:**
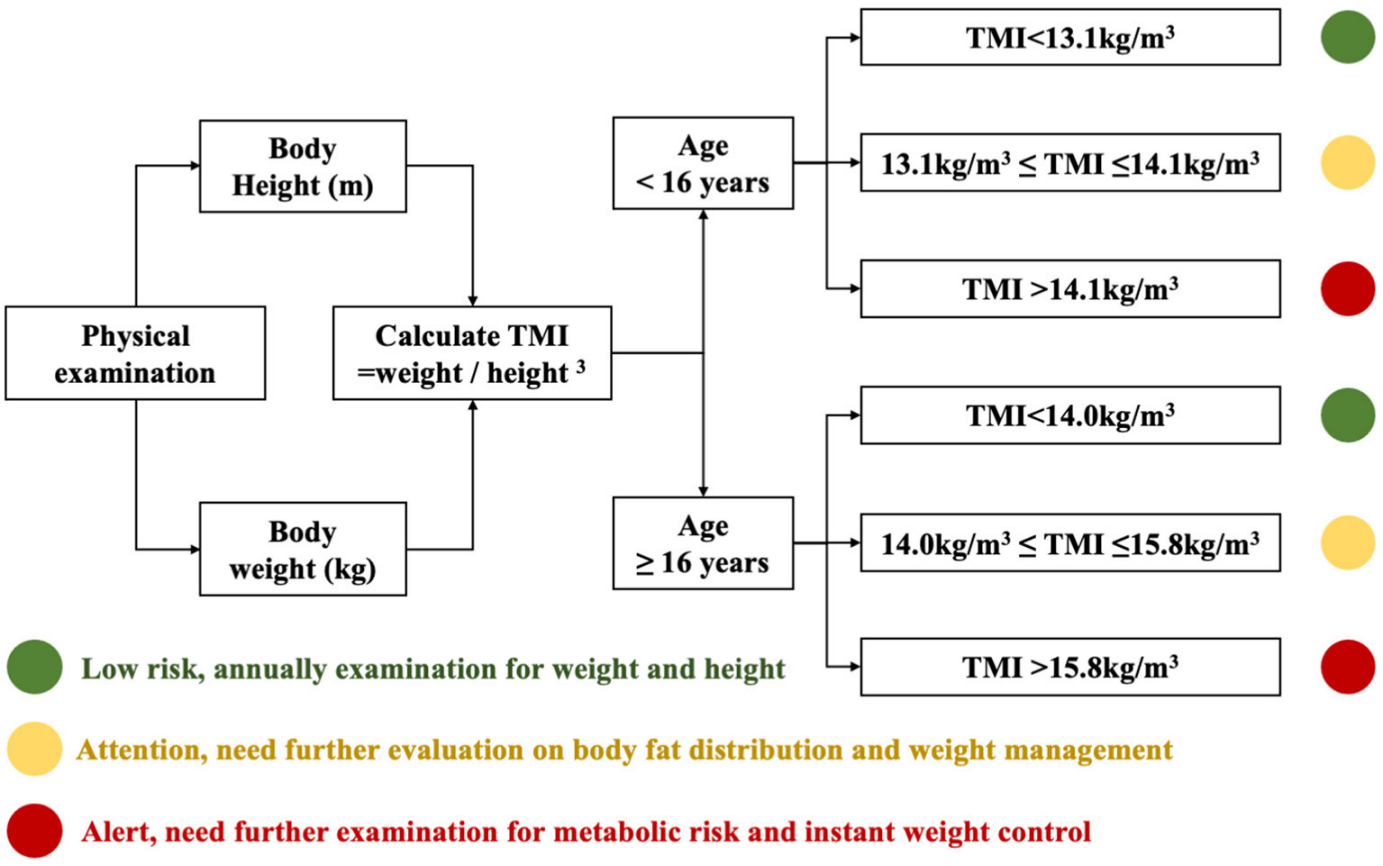
A diagram of risk screening for late adolescent overweight and obesity.

To simplify the TMI cutoffs for screening practice, the TMI cutoffs were merged across age and sex groups. The cutoffs to predict late adolescent overweight and obesity were 13.1 and 14.1 kg/m^3^, respectively, for children under the age of 16 and 14.0 kg/m^3^ and 15.8 kg/m^3^, respectively, for those aged 16 or over. All cutoffs showed satisfying ability in predicting late adolescent overweight and obesity, with the AUC >0.7 (Figure 3). The corresponding AUC of simplified TMI for children aged 7–15 ranged from 0.7315 (standard error, SE: 0.0132) to 0.9367 (SE: 0.0052). The corresponding AUC for children aged 16–18 ranged from 0.9189 (SE: 0.0048) to 0.9841 (SE: 0.0027). The AUCs of simplified TMI were significantly higher than those of BMI-WHO cutoffs, especially below the age of 15. Meanwhile, although it was not surprising that the correctly classified rate for late adolescent overweight and obesity was ≥ 90% in elder children, the performance of simplified TMI cutoffs was also satisfying in younger children. For instance, the correctly classified rates of discriminating late adolescent overweight were 80.6% for boys of age 8 years and 85.4% for girls of age 8 years. The rate of correctly discriminated obesity was even higher. The sensitivity, specificity, and correctly classified rates of simplified TMI cutoffs in discriminating overweight and obesity at late adolescence are displayed in Supplementary eTable 6. A fast risk screening diagram for late adolescent overweight and obesity was developed based on the present findings and could be updated for practical use once the findings from this study are verified with large and representative population-based studies (Figure 4).

## Discussion

This study was able to investigate if the TMI and BMI cutoffs derived from the longitudinal cohort data differed in the capability of predicting overweight and obesity at late adolescence and also to estimate whether the simplified TMI cutoffs could be easy and efficient in predicting the risk of late adolescent overweight and obesity.

In our analysis, the BMI-cohort cutoffs were generally lower than that of BMI-WHO reference and that from the Working Group on Obesity in China (9, 10). These results were similar to what N. Kartiosuo et al. had reported in their study (12). One possible explanation was that the children in the cross-sectional samples had higher BMI at the same age than their predecessors when obesity prevalence increased across the population (12). On the other hand, the cutoffs from cross-sectional data were derived based on fixed percentiles (such as P_85_ and P_95_) (9, 10), while the actual prevalence in longitudinal follow-up changed with age and peaked at the age of 11–13. The actual obesity risk in young-aged adolescents may therefore be underestimated using cutoffs derived from cross-sectional data. Therefore, national growth cohorts are valuable for estimating the age-specific percentiles.

At present, the most common way for obesity screening was to calculate either sex- and age-specific BMI z-scores or percentiles and to compare with the growth reference. Given the fact that BMI grows continuously during childhood, age-in-month-specific cutoffs were normally used. For instance, to screen overweight in children aged 7–18 years, 144 cutoffs should be referred. Altogether, there would be 576 cutoffs to screen overweight and obesity in children aged 7–18 years, which were hard to merge due to the fast development of BMI values during childhood and adolescence. Therefore, we further explored how the calculated TMI cutoffs performed in comparison with WHO and cohort-derived BMI cutoffs in terms of predicting the risk of overweight and obesity at late adolescence. The results showed that the TMI cutoffs had very similar ability compared with the BMI-cohort cutoffs and had significantly higher ability than the BMI-WHO cutoffs (especially in children under the age of 16). These calculated TMI cutoffs could be considered as a satisfying alternative for obesity risk screening in children and adolescents. Furthermore, these TMI cutoffs could be further simplified with four cutoff points covering for children aged 7–18 (two for overweight and two for obesity). Compared with the previous BMI-based screening system, the simplified TMI cutoffs could largely reduce the calculation and complexity required for overweight and obesity screening. An instructive screening diagram was also developed in our study, which could be used to assist obesity prevention and control by the greater grassroots health workforce. As the performance of simplified TMI cutoffs was especially satisfying with children under the age of 13, findings from this study support that TMI could be appropriate for early screening of overweight and obesity risk in primary schools and families with early school-aged children. However, as this study population comes from Zhongshan, Guangdong, their height and weight were at a higher level compared with the average in Guangdong province, while at a lower level compared with the national level. TMI cutoffs should be validated using a national representative sample before making clinical recommendations.

Essentially, TMI was indicative of overall body weight rather than fat distribution. It is debatable if waist circumference (WC) or waist-to-height ratio (WhtR) should be considered as primary measures in obesity screening (22, 23). However, WC or WhtR was much less used than BMI in public health practice, and the association between WC-related indicators and risk of cardiovascular disease remained inconsistent in pediatric populations (24, 25). Our previous work has also found that the combined use of TMI and WhtR did not enhance the ability in discriminating obesity and related metabolic risks (18). Furthermore, the AUCs of simplified TMI cutoffs were significantly lower than both BMI cutoffs in some late adolescence years (especially between ages 16 and 18), which may be related to the reference of BMI to define the late adolescence overweight and obese outcomes. Further research with an independent outcome measure, such as fat distribution or cardiovascular metabolic diseases, is needed to confirm the findings.

This study has a few strengths. First, for the first time, the use of TMI to predict overweight and obesity were explored in a cohort setting. The findings of this study provide (1) best-fit model equations to calculate the age- and sex-specific TMI cutoffs; (2) simplified cutoffs for obesity prediction that could be administered in public health practice. Second, we were able to make comparisons between BMI cutoffs derived from cohort data with that derived from cross-sectional data and to further verify the efficacy of TMI cutoffs. The results indicated that it was necessary to involve cohort datasets in growth references development whenever possible.

However, this study has several limitations. First, findings may not be generalizable to the nationwide student population, as the study population was from one of the 34 provinces in China. The TMI cutoffs, as well as the obesity prediction and management diagram, were in need of further validation among the various pediatric populations (26). Second, to achieve a fast-screening outcome by applying a limited number of TMI cutoffs, there may be an increased risk of misclassification. Individualized comprehensive evaluations of the overweight and obesity risks should be conducted before weight management and control practices.

## Conclusions

TMI with the ease of administration in practice could be a promising alternative screening tool to BMI for the prediction of late adolescent overweight and obesity in Chinese students.

## Data Availability Statement

The original contributions presented in the study are included in the article/Supplementary Materials, further inquiries can be directed to the corresponding author/s.

## Ethics Statement

The studies involving human participants were reviewed and approved by Institutional Review Board of Peking University. Written informed consent from the participants' legal guardian/next of kin was not required to participate in this study in accordance with the national legislation and the institutional requirements.

## Author Contributions

XW conceptualized the study, carried out the initial analyses, and drafted the initial draft. JM, BD, and YD designed the study and the data collection instruments, reviewed, and revised the manuscript. SH designed the data collection instruments, collected data, and reviewed the manuscript. ZY helped with the initial analyses, reviewed, and revised the manuscript. JH and WL critically reviewed and revised the manuscript for important intellectual content and language. All authors approved the final manuscript as submitted and agreed to be accountable for all aspects of the work.

## Funding

This work was supported by the National Natural Science Foundation (81903344 to BD and 81673192 to JM). Other authors received no external funding. The funder did not participate in the work.

## Conflict of Interest

The authors declare that the research was conducted in the absence of any commercial or financial relationships that could be construed as a potential conflict of interest.

## Publisher's Note

All claims expressed in this article are solely those of the authors and do not necessarily represent those of their affiliated organizations, or those of the publisher, the editors and the reviewers. Any product that may be evaluated in this article, or claim that may be made by its manufacturer, is not guaranteed or endorsed by the publisher.

## Abbreviations

GBD: Global Burden of Diseases, Injuries, and Risk Factors Study
BMI: body mass index
WHO: World Health Organization
TMI: tri-ponderal mass index
AUC: area under the curve
ROC curves: receiver operating characteristic curves
WC: waist circumference
WHtR: waist-to-height ratio.
