# Supplementary material for: Use of Tri-Ponderal Mass Index in Predicting Late Adolescent Overweight and Obesity in Children Aged 7–18
Source: Front Nutr. 2022 Mar 21;9:785863. doi: 10.3389/fnut.2022.785863 (PMC8978718; doi:10.3389/fnut.2022.785863)
Supplement: Supplementary file 1 [file Data_Sheet_1.docx]

| eTable 1. Possible regression models indicating the change of TMI threshold along with age. | | | | | |  |
| --- | --- | --- | --- | --- | --- | --- |
| Sex | Outcome at late adolescence | Type of regression model | Model parameters | R^2^ | Adjusted R^2^ |  |
|  |  |  |  |  |  |  |
| Boys | Overweight | Linear | y= 0.091982 x+ 12.07094 | 0.5819 | 0.5401 |  |
|  |  | Quadratic | y= -0.4068 x+ 0.02 x^2^ + 14.9507 | 0.8375 | 0.8013^*^ |  |
|  |  | Logarithmic | y= 0.9934 log(x) + 10.7525 | 0.4874 | 0.4361 |  |
|  |  | Log-linear | log(y)= 0.0068 x + 2.4959 | 0.5834 | 0.5417 |  |
|  |  | Log-log | log(y)= 0.073837 log(x) + 2.397839 | 0.4895 | 0.4384 |  |
|  | Obesity | Linear | y= 0.021888 x + 11.89887 | 0.8102 | 0.7913 |  |
|  |  | Quadratic | y= -0.3328 x + 0.0221 x^2^ +15.0841 | 0.8871 | 0.8620^*^ |  |
|  |  | Logarithmic | y= 2.4716 log(x) + 8.4938 | 0.7419 | 0.7161 |  |
|  |  | Log-linear | log(y)= 0.0147 x + 2.4977 | 0.8192 | 0.8011 |  |
|  |  | Log-log | log(y)= 0.1669 log(x) + 2.2671 | 0.7551 | 0.7307 |  |
|  |  |  |  |  |  |  |
| Girls | Overweight | Linear | y= 0.249198 x + 10.51929 | 0.9131 | 0.9044 |  |
|  |  | Quadratic | y= -0.1079 x + 0.0143 x^2^ + 12.5809 | 0.9411 | 0.9280^*^ |  |
|  |  | Logarithmic | y= 2.8360 log(x) + 6.5878 | 0.8493 | 0.8342 |  |
|  |  | Log-linear | log(y)= 0.0182 x + 2.3831 | 0.9110 | 0.9021 |  |
|  |  | Log-log | log(y)= 2.207287 log(x) + 2.095376 | 0.8502 | 0.8352 |  |
|  | Obesity | Linear | y= 0.363743 x + 10.73123 | 0.852 | 0.8372 |  |
|  |  | Quadratic | y= -0.7649 x+ 0.0451 x^2^+ 17.2474 | 0.9745 | 0.9689 |  |
|  |  | Logarithmic | y= 4.0486 log(x) + 5.2186 | 0.7580 | 0.7338 |  |
|  |  | Log-linear | log(y)= 0.0233 x + 2.4318 | 0.8676 | 0.8544 |  |
|  |  | Log-log | log(y)= 0.2598 log(x) + 2.0771 | 0.7772 | 0.7549 |  |
| TMI, tri-ponderal mass index, kg/m^3^. ^*^ Best fit model according to adjusted R^2^. Y was TMI cutoff, and x was age. | | | | | |  |

| eTable 2. Age and sex specific percentiles of tri-ponderal mass index (TMI) in children aged 7 to 18. | | | | | | | | | | | |
| --- | --- | --- | --- | --- | --- | --- | --- | --- | --- | --- | --- |
| Sex | Age | TMI Percentiles (kg/m^3^) | | | | | | | | | |
|  |  | P_3_ | P_10_ | P_25_ | P_50_ | P_75_ | P_85_ | P_90_ | P_95_ | P_97_ |  |
| Boys |  |  |  |  |  |  |  |  |  |  |  |
|  | 7 | 10.57 | 11.10 | 11.70 | 12.47 | 13.38 | 13.86 | 14.38 | 15.36 | 15.60 |  |
|  | 8 | 10.25 | 10.81 | 11.46 | 12.29 | 13.31 | 13.91 | 14.45 | 15.64 | 15.89 |  |
|  | 9 | 9.93 | 10.53 | 11.22 | 12.13 | 13.26 | 14.13 | 14.57 | 15.98 | 16.28 |  |
|  | 10 | 9.66 | 10.28 | 11.01 | 11.99 | 13.23 | 14.36 | 14.71 | 16.33 | 16.72 |  |
|  | 11 | 9.46 | 10.10 | 10.85 | 11.88 | 13.19 | 14.38 | 14.79 | 16.40 | 17.02 |  |
|  | 12 | 9.37 | 10.01 | 10.76 | 11.78 | 13.10 | 14.21 | 14.71 | 16.15 | 16.96 |  |
|  | 13 | 9.35 | 9.97 | 10.71 | 11.71 | 12.98 | 13.91 | 14.53 | 15.94 | 16.66 |  |
|  | 14 | 9.35 | 9.96 | 10.68 | 11.65 | 12.89 | 13.74 | 14.37 | 15.70 | 16.40 |  |
|  | 15 | 9.35 | 9.96 | 10.67 | 11.64 | 12.86 | 13.76 | 14.34 | 15.74 | 16.34 |  |
|  | 16 | 9.36 | 9.97 | 10.70 | 11.68 | 12.92 | 13.98 | 14.42 | 15.86 | 16.47 |  |
|  | 17 | 9.39 | 10.01 | 10.75 | 11.75 | 13.02 | 14.08 | 14.57 | 16.02 | 16.70 |  |
|  | 18 | 9.43 | 10.06 | 10.82 | 11.83 | 13.14 | 14.20 | 14.73 | 16.23 | 16.94 |  |
| Girls |  |  |  |  |  |  |  |  |  |  |  |
|  | 7 | 10.22 | 10.77 | 11.38 | 12.13 | 13.00 | 13.50 | 14.00 | 14.69 | 15.25 |  |
|  | 8 | 9.87 | 10.44 | 11.07 | 11.85 | 12.78 | 13.33 | 13.86 | 14.85 | 15.23 |  |
|  | 9 | 9.55 | 10.13 | 10.78 | 11.60 | 12.58 | 13.30 | 13.74 | 14.84 | 15.26 |  |
|  | 10 | 9.29 | 9.88 | 10.56 | 11.41 | 12.45 | 13.17 | 13.69 | 14.85 | 15.36 |  |
|  | 11 | 9.16 | 9.77 | 10.47 | 11.35 | 12.45 | 13.25 | 13.77 | 14.98 | 15.57 |  |
|  | 12 | 9.23 | 9.85 | 10.56 | 11.48 | 12.61 | 13.56 | 13.99 | 15.28 | 15.88 |  |
|  | 13 | 9.45 | 10.09 | 10.82 | 11.74 | 12.89 | 13.90 | 14.28 | 15.59 | 16.18 |  |
|  | 14 | 9.72 | 10.36 | 11.09 | 12.02 | 13.17 | 14.09 | 14.55 | 15.78 | 16.42 |  |
|  | 15 | 9.92 | 10.57 | 11.30 | 12.23 | 13.37 | 14.11 | 14.73 | 15.76 | 16.56 |  |
|  | 16 | 10.03 | 10.67 | 11.41 | 12.33 | 13.47 | 14.19 | 14.82 | 15.92 | 16.63 |  |
|  | 17 | 10.07 | 10.71 | 11.45 | 12.37 | 13.50 | 14.18 | 14.85 | 15.82 | 16.65 |  |
|  | 18 | 10.09 | 10.73 | 11.46 | 12.38 | 13.50 | 14.14 | 14.84 | 15.60 | 16.63 |  |

| eTable 3. Best fit BMI and TMI cutoffs for late adolescence overweight and obesity or obesity only. | | | | | | | | | |  |
| --- | --- | --- | --- | --- | --- | --- | --- | --- | --- | --- |
| Age | Boys | | | |  | Girls | | | |  |
|  | Overweight & obesity | | Obesity | |  | Overweight & obesity | | Obesity | |  |
|  | BMI | TMI | BMI | TMI |  | BMI | TMI | BMI | TMI |  |
| 7 | 15.98 | 12.95 | 16.80 | 13.66 |  | 15.95 | 12.74 | 17.43 | 13.99 |  |
| 8 | 16.77 | 12.83 | 17.78 | 13.67 |  | 16.61 | 12.82 | 18.90 | 14.23 |  |
| 9 | 17.28 | 13.03 | 19.08 | 14.07 |  | 17.38 | 12.54 | 19.06 | 14.14 |  |
| 10 | 18.38 | 13.14 | 20.19 | 14.08 |  | 17.98 | 12.49 | 20.04 | 13.78 |  |
| 11 | 19.27 | 13.12 | 21.13 | 14.58 |  | 19.30 | 12.99 | 21.45 | 14.27 |  |
| 12 | 20.17 | 12.90 | 21.91 | 14.47 |  | 20.51 | 13.31 | 22.71 | 14.63 |  |
| 13 | 21.10 | 13.00 | 23.69 | 14.34 |  | 21.42 | 13.70 | 24.60 | 15.08 |  |
| 14 | 21.74 | 12.95 | 24.08 | 14.13 |  | 22.21 | 14.14 | 24.75 | 15.29 |  |
| 15 | 22.32 | 13.05 | 24.97 | 14.77 |  | 22.89 | 14.32 | 25.15 | 16.30 |  |
| 16 | 23.56 | 13.56 | 26.68 | 15.51 |  | 23.26 | 14.73 | 26.41 | 16.30 |  |
| 17 | 24.31 | 13.94 | 28.14 | 16.06 |  | 24.30 | 14.83 | 28.89 | 17.00 |  |
| 18 | 24.96 | 14.17 | 29.34 | 16.29 |  | 24.88 | 15.01 | 30.43 | 18.34 |  |
| BMI, body mass index, kg/m^2^; TMI, tri-ponderal mass index, kg/m^3^. | | | | | | | | | |  |
|  |  |  |  |  |  |  |  |  |  |  |

| eTable 4. The area under curves of best-fit TMI cutoffs in discriminating overweight and obesity at late adolescence | | | | | | | | | |
| --- | --- | --- | --- | --- | --- | --- | --- | --- | --- |
| Age | Overweight | | | |  | Obesity | | | |
|  | Cutoffs | Sensitivity | Specificity | Area under curve (95% CI) |  | Cutoffs | Sensitivity | Specificity | Area under curve (95% CI) |
| Boys |  |  |  |  |  |  |  |  |  |
| 7 | 12.95 | 73.7% | 74.5% | 0.802 (0.785, 0.817) |  | 13.66 | 83.3% | 84.1% | 0.894 (0.882, 0.906) |
| 8 | 12.83 | 79.7% | 76.8% | 0.862 (0.851, 0.872) |  | 13.67 | 86.1% | 84.6% | 0.923 (0.914, 0.931) |
| 9 | 13.03 | 80.7% | 81.3% | 0.886 (0.878, 0.894) |  | 14.07 | 87.9% | 86.9% | 0.933 (0.927, 0.939) |
| 10 | 13.14 | 83.1% | 81.8% | 0.901 (0.894, 0.908) |  | 14.08 | 90.8% | 85.1% | 0.938 (0.933, 0.944) |
| 11 | 13.12 | 83.6% | 81.7% | 0.906 (0.899, 0.912) |  | 14.58 | 88.2% | 89.2% | 0.945 (0.940, 0.950) |
| 12 | 12.90 | 85.1% | 81.6% | 0.910 (0.904, 0.916) |  | 14.47 | 90.5% | 89.5% | 0.958 (0.953, 0.962) |
| 13 | 13.00 | 84.2% | 85.8% | 0.926 (0.920, 0.931) |  | 14.34 | 92.8% | 90.8% | 0.969 (0.965, 0.972) |
| 14 | 12.95 | 87.4% | 87.2% | 0.942 (0.937, 0.947) |  | 14.13 | 94.8% | 90.7% | 0.973 (0.970, 0.977) |
| 15 | 13.05 | 90.8% | 88.5% | 0.957 (0.953, 0.961) |  | 14.77 | 94.3% | 94.3% | 0.984 (0.981, 0.986) |
| 16 | 13.56 | 93.7% | 93.1% | 0.982 (0.979, 0.985) |  | 15.51 | 96.6% | 96.9% | 0.994 (0.992, 0.996) |
| 17 | 13.94 | 95.0% | 94.9% | 0.989 (0.986, 0.992) |  | 16.06 | 97.9% | 98.3% | 0.996 (0.994, 0.998) |
| 18 | 14.17 | 84.5% | 99.3% | 0.994 (0.988, 0.997) |  | 16.29 | 97.7% | 98.2% | 0.998 (0.994, 1.000) |
| Girls |  |  |  |  |  |  |  |  |  |
| 7 | 12.74 | 83.4% | 75.8% | 0.869 (0.854, 0.883) |  | 13.99 | 89.5% | 91.6% | 0.952 (0.942, 0.961) |
| 8 | 12.82 | 83.5% | 81.8% | 0.898 (0.888, 0.908) |  | 14.23 | 88.0% | 93.1% | 0.955 (0.947, 0.961) |
| 9 | 12.54 | 88.7% | 79.6% | 0.91 (0.902, 0.917) |  | 14.14 | 92.3% | 92.9% | 0.967 (0.962, 0.972) |
| 10 | 12.49 | 86.5% | 81.5% | 0.917 (0.91, 0.923) |  | 13.78 | 93.6% | 90.9% | 0.97 (0.966, 0.974) |
| 11 | 12.99 | 87.0% | 86.7% | 0.939 (0.934, 0.945) |  | 14.27 | 96.3% | 93.0% | 0.979 (0.976, 0.982) |
| 12 | 13.31 | 89.0% | 87.5% | 0.949 (0.943, 0.954) |  | 14.63 | 95.5% | 93.6% | 0.986 (0.983, 0.988) |
| 13 | 13.70 | 90.6% | 88.1% | 0.957 (0.952, 0.961) |  | 15.08 | 95.5% | 94.1% | 0.982 (0.979, 0.985) |
| 14 | 14.14 | 89.2% | 90.7% | 0.964 (0.959, 0.968) |  | 15.29 | 94.6% | 94.4% | 0.985 (0.982, 0.987) |
| 15 | 14.32 | 92.0% | 92.1% | 0.972 (0.968, 0.975) |  | 16.30 | 95.1% | 97.3% | 0.99 (0.988, 0.992) |
| 16 | 14.73 | 93.6% | 95.2% | 0.986 (0.984, 0.989) |  | 16.30 | 98.9% | 97.0% | 0.996 (0.995, 0.998) |
| 17 | 14.83 | 96.5% | 95.6% | 0.993 (0.99, 0.995) |  | 17.00 | 100.0% | 98.6% | 0.999 (0.997, 1) |
| 18 | 15.01 | 98.0% | 96.8% | 0.995 (0.988, 0.998) |  | 18.34 | 100.0% | 99.9% | 1 (0.996, 1) |

| eTable 5. Calculated age-specific TMI cutoffs to discriminate overweight and obesity or obesity at late adolescence | | | | | |
| --- | --- | --- | --- | --- | --- |
| Age | Boys | |  | Girls | |
|  | Overweight & obesity | Obesity |  | Overweight & obesity | Obesity |
| 7~ | 13.02 | 13.83 |  | 12.58 | 14.05 |
| 8~ | 12.94 | 13.85 |  | 12.70 | 14.01 |
| 9~ | 12.89 | 13.92 |  | 12.85 | 14.06 |
| 10~ | 12.89 | 14.03 |  | 13.03 | 14.19 |
| 11~ | 12.92 | 14.18 |  | 13.23 | 14.42 |
| 12~ | 12.99 | 14.37 |  | 13.46 | 14.73 |
| 13~ | 13.10 | 14.61 |  | 13.72 | 15.13 |
| 14~ | 13.25 | 14.90 |  | 14.01 | 15.63 |
| 15~ | 13.44 | 15.21 |  | 14.32 | 16.18 |
| 16~ | 13.67 | 15.59 |  | 14.68 | 16.87 |
| 17~ | 13.93 | 15.99 |  | 15.04 | 17.60 |
| 18~ | 14.19 | 16.38 |  | 15.37 | 18.31 |

| eTable 6. The sensitivity, specificity and correct classification rate of simplified TMI cutoffs in discriminating overweight and obesity at late adolescence. | | | | | | | | | | | | | |
| --- | --- | --- | --- | --- | --- | --- | --- | --- | --- | --- | --- | --- | --- |
| Weight status at end point | Age | Total | | |  | Boys | | |  | Girls | | | |
|  |  | Sensitivity | Specificity | Correctly classified |  | Sensitivity | Specificity | Correctly classified |  | Sensitivity | Specificity | Correctly classified |  |
| Overweight | 7 | 69.22% | 80.10% | 78.86% |  | 68.56% | 77.74% | 76.40% |  | 70.70% | 82.65% | 81.74% |  |
|  | 8 | 74.51% | 83.74% | 82.76% |  | 73.88% | 81.61% | 80.56% |  | 76.03% | 86.08% | 85.40% |  |
|  | 9 | 78.41% | 84.51% | 83.87% |  | 79.46% | 82.23% | 81.85% |  | 75.77% | 86.97% | 86.24% |  |
|  | 10 | 81.65% | 84.73% | 84.41% |  | 83.59% | 81.18% | 81.50% |  | 77.08% | 88.54% | 87.78% |  |
|  | 11 | 83.65% | 84.64% | 84.54% |  | 83.73% | 81.55% | 81.85% |  | 83.44% | 87.93% | 87.65% |  |
|  | 12 | 84.19% | 84.33% | 84.32% |  | 81.55% | 84.03% | 83.69% |  | 90.70% | 84.65% | 85.04% |  |
|  | 13 | 86.41% | 83.47% | 83.77% |  | 82.70% | 86.76% | 86.21% |  | 95.67% | 79.93% | 80.93% |  |
|  | 14 | 88.68% | 82.27% | 82.93% |  | 85.17% | 89.00% | 88.48% |  | 97.29% | 75.03% | 76.46% |  |
|  | 15 | 92.17% | 81.65% | 82.73% |  | 89.80% | 89.02% | 89.13% |  | 98.14% | 73.70% | 75.25% |  |
|  | 16 | 90.52% | 92.55% | 92.34% |  | 87.26% | 96.52% | 95.26% |  | 98.44% | 88.29% | 88.94% |  |
|  | 17 | 95.40% | 91.86% | 92.19% |  | 93.71% | 95.37% | 95.17% |  | 99.65% | 87.99% | 88.67% |  |
|  | 18 | 98.53% | 91.04% | 91.67% |  | 98.06% | 93.67% | 94.17% |  | 100.00% | 87.88% | 88.44% |  |
| Obesity | 7 | 43.73% | 93.66% | 87.98% |  | 43.63% | 92.73% | 85.55% |  | 43.95% | 94.67% | 90.82% |  |
|  | 8 | 54.85% | 93.64% | 89.55% |  | 54.47% | 92.69% | 87.45% |  | 55.79% | 94.70% | 92.05% |  |
|  | 9 | 59.95% | 93.51% | 89.99% |  | 60.94% | 92.08% | 87.74% |  | 57.46% | 95.06% | 92.62% |  |
|  | 10 | 64.17% | 92.78% | 89.84% |  | 67.27% | 90.55% | 87.42% |  | 56.85% | 95.17% | 92.63% |  |
|  | 11 | 66.60% | 92.71% | 90.02% |  | 66.40% | 90.61% | 87.28% |  | 67.11% | 94.94% | 93.17% |  |
|  | 12 | 67.48% | 93.15% | 90.50% |  | 64.36% | 92.24% | 88.39% |  | 75.21% | 94.13% | 92.91% |  |
|  | 13 | 70.29% | 93.30% | 90.94% |  | 64.85% | 94.53% | 90.48% |  | 83.86% | 91.99% | 91.47% |  |
|  | 14 | 71.93% | 93.16% | 90.99% |  | 64.83% | 95.80% | 91.62% |  | 89.36% | 90.31% | 90.25% |  |
|  | 15 | 76.50% | 93.51% | 91.75% |  | 69.82% | 96.54% | 92.88% |  | 93.40% | 90.24% | 90.44% |  |
|  | 16 | 46.91% | 99.55% | 94.14% |  | 37.18% | 99.90% | 91.39% |  | 70.57% | 99.18% | 97.32% |  |
|  | 17 | 54.00% | 99.60% | 95.35% |  | 45.45% | 99.90% | 93.21% |  | 75.44% | 99.27% | 97.89% |  |
|  | 18 | 60.29% | 99.68% | 96.34% |  | 56.13% | 100.00% | 94.98% |  | 73.47% | 99.30% | 98.09% |  |


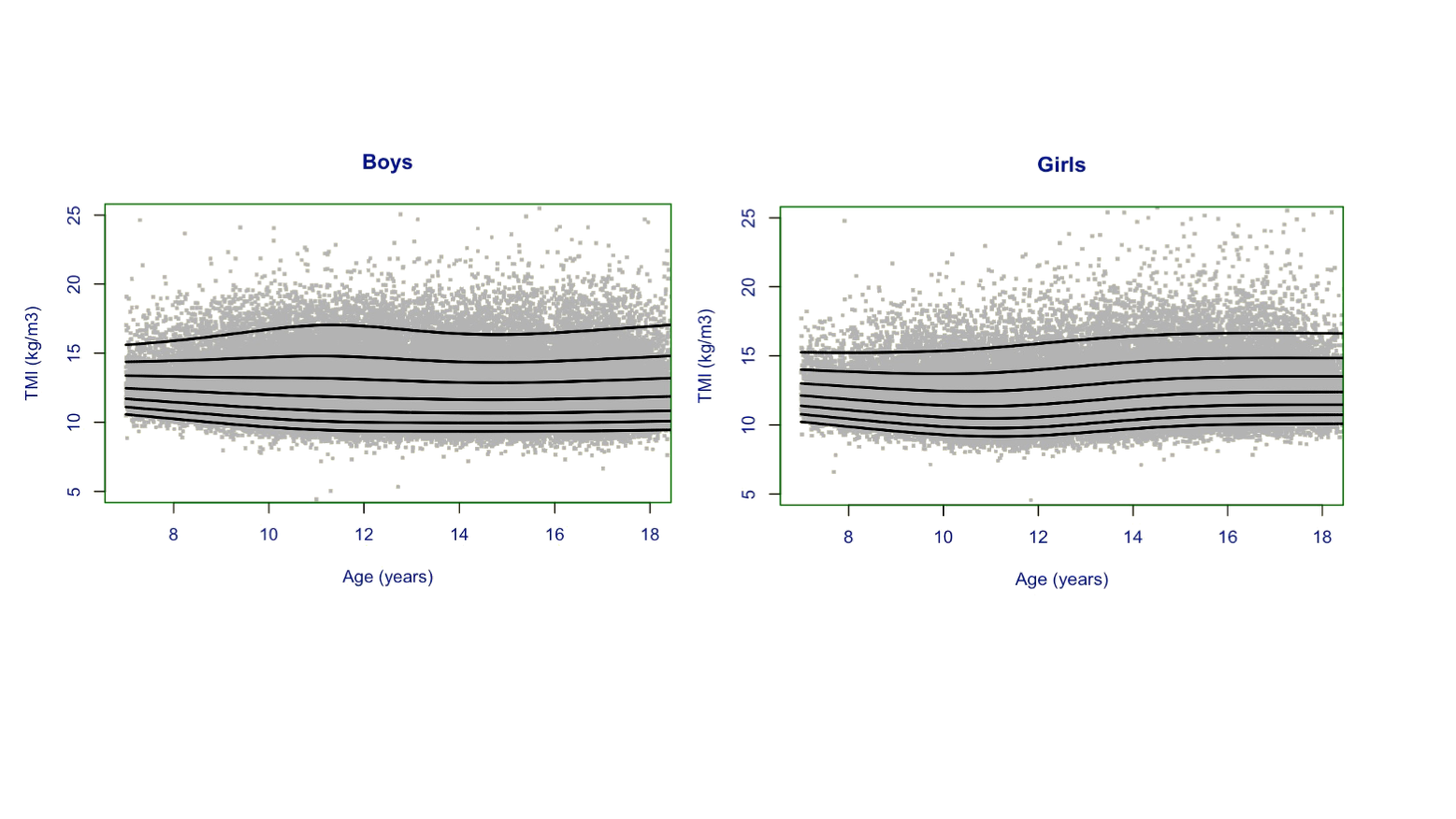


eFigure 1. Tri-ponderal mass index (TMI) values and age-specific percentiles in both boys and girls.

The curves were P_3_, P_10_, P_25_, P_50_, P_75_, P_90_ and P_97_ from bottom to top, respectively.


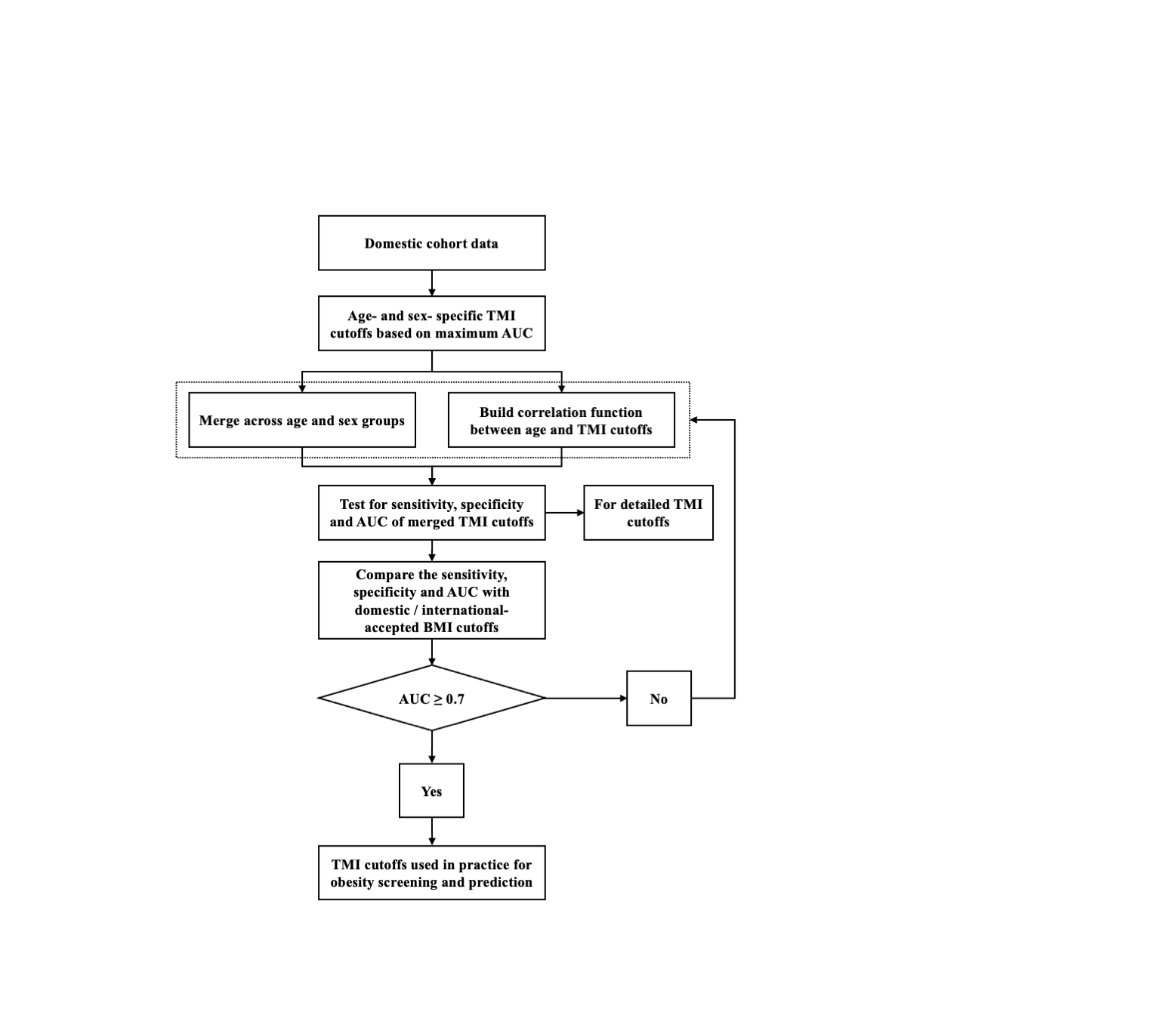


eFigure 2. Process of identifying the tri-ponderal mass index (TMI) cutoffs from the domestic cohort data.

AUC, area under curve.
